# Vilazodone Alleviates Neurogenesis-Induced Anxiety in the Chronic Unpredictable Mild Stress Female Rat Model: Role of Wnt/β-Catenin Signaling

**DOI:** 10.1007/s12035-024-04142-3

**Published:** 2024-04-08

**Authors:** Rana A. El-Kadi, Noha F. AbdelKader, Hala F. Zaki, Ahmed S. Kamel

**Affiliations:** 1 Pharmacology and Toxicology Department, Faculty of Pharmacy, Cairo University, Kasr El-Aini, Cairo, 11562 Egypt; 2 Alexandria University Hospitals, Champollion Street, El-Khartoum Square, El Azareeta, Alexandria, 21131 Egypt

**Keywords:** Vilazodone, Anxiety, Stress, Neurogenesis, Wnt/β-catenin, MicroRNAs

## Abstract

Defective β-catenin signaling is accompanied with compensatory neurogenesis process that may pave to anxiety. β-Catenin has a distinct role in alleviating anxiety in adolescence; however, it undergoes degradation by the degradation complex Axin and APC. Vilazodone (VZ) is a fast, effective antidepressant with SSRI activity and 5-HT_1A_ partial agonism that amends somatic and/or psychic symptoms of anxiety. Yet, there is no data about anxiolytic effect of VZ on anxiety-related neurogenesis provoked by stress-reduced β-catenin signaling. Furthermore, females have specific susceptibility toward psychopathology. The aim of the present study is to uncover the molecular mechanism of VZ relative to Wnt/β-catenin signaling in female rats. Stress-induced anxiety was conducted by subjecting the rats to different stressful stimuli for 21 days. On the 15th day, stressed rats were treated with VZ(10 mg/kg, p.o.) alone or concomitant with the Wnt inhibitor: XAV939 (0.1 mg/kg, i.p.). Anxious rats showed low β-catenin level turned over by Axin-1 with unanticipated reduction of APC pursued with elevated protein levels of neurogenesis-stimulating proteins: c-Myc and p^Thr183^-Erk likewise gene expressions of miR-17-5p and miR-18. Two weeks of VZ treatment showed anxiolytic effect figured by alleviation of hippocampal histological examination. VZ protected β-catenin signal via reduction in Axin-1 and elevation of APC conjugated with modulation of β-catenin downstream targets. The cytoplasmic β-catenin turnover by Axin-1 was restored by XAV939. Herein, VZ showed anti-anxiety effect, which may be in part through regaining the balance of the reduced β-catenin and its subsequent exaggerated response of p-Erk, c-Myc, Dicer-1, miR-17-5p, and miR-18.

**Graphical Abstract:**

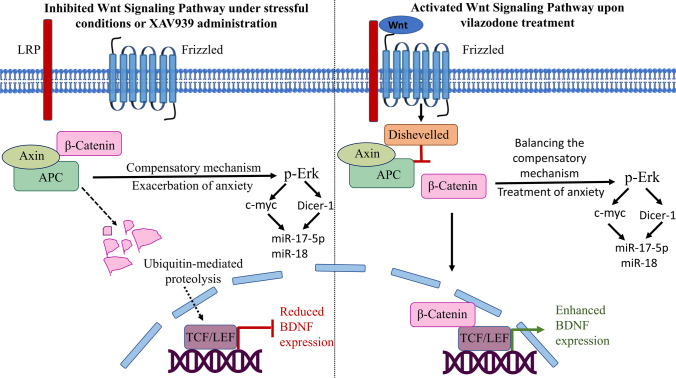

## Introduction

Stress is an unavoidable aspect of human life. Unfortunately, stressful life events may cause pathological status like psychiatric disorders [1]. Anxiety and depression are two of the most common mental conditions that affect individuals of all ages [2]. In addition, anxiety is the most frequent outcome of psychopathology that causes chronic permanent illness [3]. Together, female gender is considered a high-risk factor for anxiety [4]. The prevalence rates of anxiety disorders in females were almost twice that found for males when data were compared across different countries [5]. One out of every five teenagers had clinically increased anxiety symptoms [6]. Furthermore, anxiety costs the global economy one trillion dollars annually, according to the World Health Organization [7].

In humans, the increased exposure to stressors during adolescence is accompanied with depression and anxiety [8]. On the other hand, adolescent stress influences adaptation techniques for dealing with future stressors in adulthood [9]. Several proteins figured out these adaptation techniques in part through c-Myc, Dicer-1, and microRNAs (miRNAs) by increasing the neurogenesis threshold [10–12]. Persistence of these changes, however, can result in high levels of stress for the adolescents, which in turn can set the stage for psychopathology [13]. MicroRNAs are noncoding RNAs with multiple functions during development and pathogenesis of different disorders [14]. MicroRNAs are characterized by an additional epigenomic regulation where they control multi-degree gene expression in the limbic circuit during early life and adulthood stresses, and hence regulate stress-related diseases [15]. Moreover, miRNAs are highly connected with anxiety [16]. One of these miRNAs is miR17-92, which is a large family comprising six miRNAs including miR-17 and miR-18 [17]. Regarding miR-17-5p, it plays an important role in cell processes such as cell proliferation, migration, and apoptosis [18]. Together, chronic stress elevates miR-18 in adult rat. On the contrary, miR-18 is considered a potential element in the development of chronic stress [19].

A polyvalent protein, β-catenin, is involved in the development of the brain proliferation and dendritic growth [20]. It is considered as a widespread target for the management of teenager stress [21]. Intriguingly, Wnt/β-catenin signaling is crucial in anxiety-related behavior where it protects against anxiety [22, 23]. β-Catenin is regulated by a destructive complex of Axin-1 and adenomatous polyposis coli (APC) [24]. β-Catenin implements its prementioned roles through several downstream targets namely, T cell factor (TCF), brain-derived neurotrophic factor (BDNF), c-Myc, and Dicer-1 [25–27]. Furthermore, β-catenin/TCF pathway regulated the proliferation of adult hippocampal neural progenitor cells [28]. In addition, Dicer-1 deficiency in the central amygdala increased anxiety-like behavior in mice [29].

Chronic unpredictable mild stress (CUMS) is a well-known stress model [30]. It causes dysregulation of neurocircuits that precipitates anxiety in different brain areas [31]. The model simulates the stresses applied to adolescents and presents the pathophysiological changes that led to anxiety [32]. Unfortunately, the exact pathways and intracellular neurotransmitter’s mechanisms of anxiety are largely obscure [33]. Consequently, the mechanism that restores dysregulated neurocircuit requires novel therapeutic target for anxiety treatment.

Vilazodone (VZ) has a higher remission rate with faster onset of action than pre-existing selective serotonin reuptake inhibitor (SSRI) drugs. In addition, VZ has less adverse event risks of cardiovascular toxicity and weight gain. Consequently, VZ is safe with long-term effectiveness [34]. Vilazodone is a novel drug with dual mechanism, where it combines serotonin partial agonists and reuptake inhibitor actions [35]. This mechanism influences its power to treat somatic and physical manifestations of stress. Clinically, VZ has a potential role in the treatment of anxiety in patients suffering from depression [36]. However, so far, according to Ziffra [37], VZ has not received the US Food and Drug Administration approval for the treatment of anxiety.

Downregulation of β-catenin signal enhances neurogenesis adaptive technique that enhances the onset of anxiety [38–40], yet no information is available on effective drugs that modulate this compensatory mechanism on anxiety. Herein, the present study endeavors to clarify more about the signal transduction of VZ in dealing with the mal-adaptation response that is secondary to the diminished Wnt/β-catenin signaling under the influence of CUMS adolescent rat model. To outline this approach, the study utilized XAV939 as an inhibitor to β-catenin signaling to assure VZ effect.

## Materials and Methods

### Animals

Female Wistar Albino rats (weighing 150–180 g, 6 weeks age) were obtained from the animal facility of Faculty of Pharmacy, Cairo University (Cairo, Egypt). Rats were kept under standard housing conditions: humidity (60 ± 10%) and room temperature (25 ± 2 °C). Animals were given standard laboratory diet that allowed free access to water.

### Experimental Protocol

**Fig. 1:**
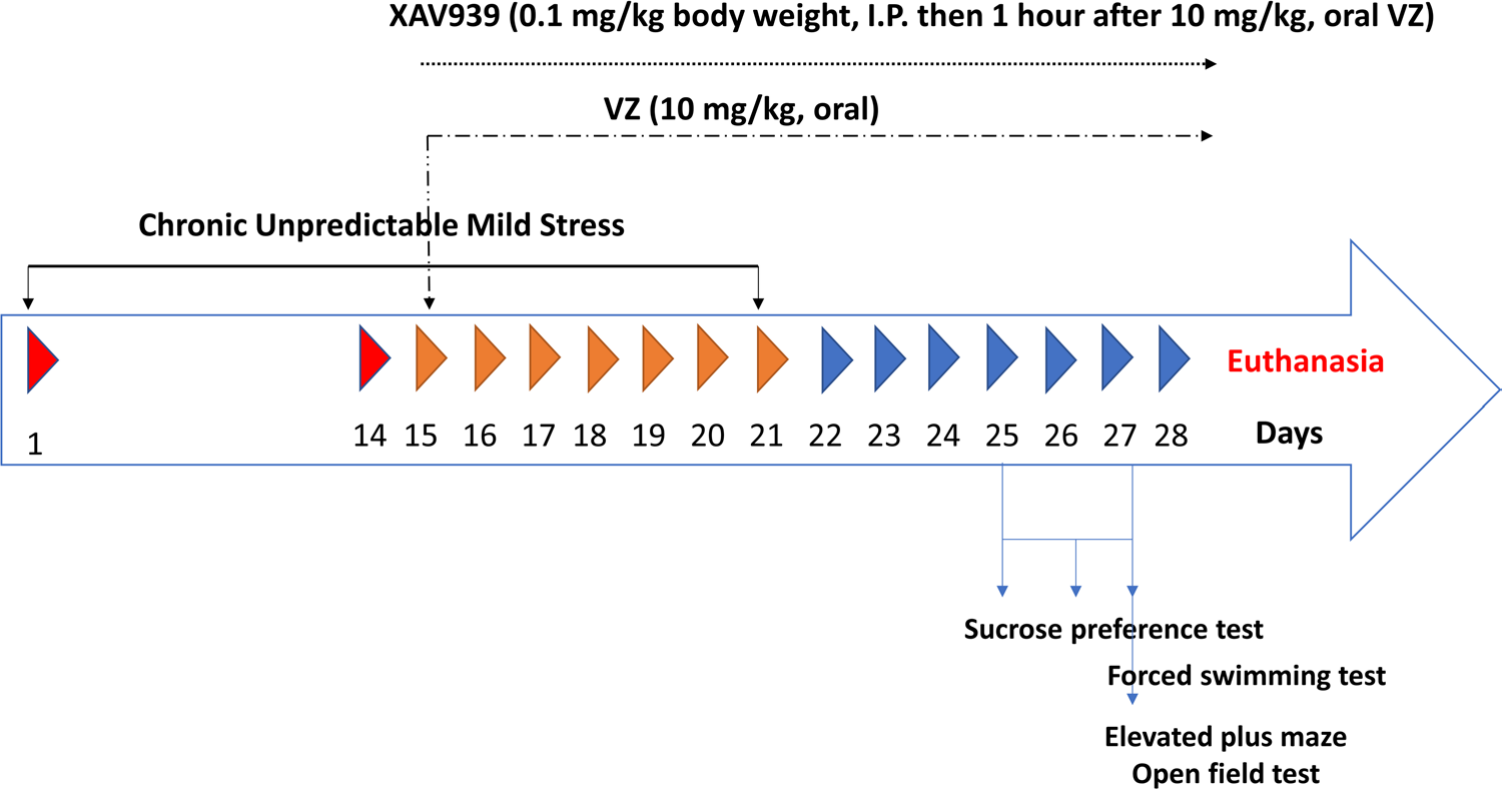
Timeline for the experiment

Forty rats were randomly divided into four experimental groups (each group contains 10 rats). Group I (CTRL) served as control and received normal saline. Group II (CUMS) was subjected to CUMS protocol and received normal saline. Group III (CUMS + VZ) was subjected to CUMS then treated with VZ (10 mg/kg, p.o.) for 2 weeks starting from day 15 after 2 weeks of stress procedures to test VZ on the already stressed animal [41–43]. Group IV (CUMS + XAV939 + VZ) was subjected to CUMS then treated with XAV939 (0.1 mg/kg, i.p.), 1 h before VZ administration to investigate the effect of the blockage of β-catenin upon VZ administration and whether this pathway is managed by VZ or not [44]. Chronic unpredictable mild stress was conducted by subjecting rats to different stressors over 21 days (Table 1) [45]. The stress procedures were conducted at the adolescence period of rats at approximately 6 weeks age [46]. All stressed rats were evaluated at around 10 weeks of age for depression by sucrose preference test (SPT) on days 25–27 and forced swimming test (FST) on day 27 as well as for anxiety by elevated plus maze (EPM) and open field test (OFT) on day 27. The testing order began with the least stressful test: SPT, OFT, followed by EPM, and then finally ended with the most stressful one, forced swimming test. The time interval between each two tests was 2 h. Timeline for the experiment is described in Fig. 1.

**Table 1:** Chronic unpredictable stress protocol

Days	Stress protocol
1, 8, and 15	Animals were put in covered transparent customized non-movable plastic jars in the fridge for 1 h at 4 ºC, then they were forced to swim in deep non-reachable bottom water bath with less than half filling with water which accounts for a high wall that prevents escaping (130 cm in diameter and 40 cm deep) for 10–15 min at 25 ºC
2, 9, and 16	Animals were put in covered transparent customized non-movable plastic jars for 1 h at 25 ºC, then each two groups were put 23 h in a small box for crowding; each box was covered with upper mesh for breathing and prevention of escaping (380 × 275 × 170 mm)
3, 10, and 17	Animals were immersed in deep non-reachable bottom ice bath with less than half filling with water which accounts for a high wall that prevents escaping (130 cm in diameter and 40 cm deep) for 5 min at 10 ºC, then they were put in a plastic-covered tube; tubes were with different diameters. Each is used to be fit with animal size and make animal non-escapable in addition to open mesh for breathing and outlet for freely moving tail for 23 h at 25 ºC
4, 11, and 18	Animals were put in covered transparent customized non-movable plastic jars for 1 h at 25 ºC, and then they were shaken for 1 h by orbital shaker
5, 12, and 19	Animals were forced to swim deep non-reachable bottom in water bath with less than half filling with water which accounts for a high wall that prevents escaping (130 cm in diameter and 40 cm deep) for 10–15 min at 25 ºC, then they were put in covered transparent customized non-movable plastic jars for 1 h in the fridge at 4 ºC
6, 13, and 20	Animals were immersed in deep non-reachable bottom ice bath with less than half filling with water which accounts for a high wall that prevents escaping (130 cm in diameter and 40 cm deep) for 5 min at 10 ºC, then each two groups were put in 23 h in a small box for crowding; each box was covered with upper mesh for breathing and prevention of escaping (380 × 275 × 170 mm)
7, 14, and 21	Animals were shaken for 1 h by orbital shaker, then they were put in a plastic covered tube; tubes were with different diameters. Each is used to be fit with animal size and make animal non-escapable in addition to open mesh for breathing and outlet for freely moving tail for 23 h at 25 ºC

### Sucrose Preference Test

The test is used to evaluate anhedonia in rodents which is a key feature of depressive symptoms. Anhedonia was assessed by assessing animal’s preference for a sweetened drink over plain drinking water. This test was carried out in the rat home cage by placing one animal per cage. Rat cages were randomly allocated with two identical bottles, one bottle containing 200-ml plain drinking water while the second containing 200-ml 1–2%w/v sucrose solution for 3 consecutive days. The 2 bottles’ positions were switched daily to avoid any side bias. After testing, cumulative consumption of sucrose was calculated, and sucrose preference was estimated for each rat using the following formula [47]:

$$\%\;Sucrose\;preference\;=\frac{sucrose\;intake}{water\;intake\;+\;sucrose\;intake\;}\times\;100$$

### Forced Swimming Test

The test is used as a depressive indicator to reflect the behavioral despair state of rats. Individual rats were placed in a transparent Plexiglas cylinder (20 cm diameter 50 cm high) filled to a depth of 30 cm with water (23–25 °C) and allowed to swim for 6 min. Rats that floated without swimming in order to keep their heads above water were considered immobile [48].

### Elevated Plus Maze Test

The EPM test was used to evaluate anxiety-like behavior. It is composed of a cross-shaped platform, two walled arms (closed arms 25 × 5 × 16 cm), two arms without walls (open arms 25 × 5 × 0.5 cm), and a middle platform (5 × 5 × 0.5 cm). The open and closed arms were directly in opposition to one another. The platforms were raised to a height of 60 cm above the ground, and the apparatus was cleaned with a 70% alcohol, and then dried with a cloth between sessions. During the sessions, the level of illumination in the behavioral lab was set to 400 lx. Each rat was placed on the middle platform, and their movements in the EPM were recorded for 5 min and evaluated using ANY-maze video tracking software Version 6.3 (Stoelting Co., Illinois, USA). Anxiety index was calculated by the following equation:

$$Anxiety\;index=1-\left[\frac{\left(\frac{time\;spend\;in\;open\;arms}{total\;time\;to\;the\;maze}\right)+\left(\frac{number\;of\;entries\;to\;the\;open\;arms}{total\;exploration\;to\;the\;maze}\right)}2\right]$$

Anxiety index values range from 0 to 1 where an increase in the index expresses increased anxiety-like behavior. In addition, open arm time, closed arm time, central zone entries, and total distance through open, closed, and central regions were recorded [49].

### Open Field Test

The OFT was carried out to assess rodents’ exploratory behavior and general activity. A square wooden box (80 × 80 × 40 cm) was used for the experiment. Each rat was gently placed in the center of the open field and allowed to freely explore the area for 5 min before the floor was cleaned. ANY-Maze video tracking software Version 6.3 (Stoelting Co., Illinois, USA) was used to analyze rat’s behavior that was recorded with a video camera placed on top of the box. Rearing and central distance were recorded. The animal’s position in the periphery indicates anxiety. Its tendency toward the center, on the other hand, indicates anti-stress action [50].

### Brain Processing

One week after CUMS (day 28), rats were euthanized by decapitation under anesthesia. Consequently, animals’ brains were collected, washed, dried, and weighted. Animal’s brains were allocated into two subsets for each group. First subset (*n* = 3), rat’s brains were prepared for histological and Ki-67 immunohistochemistry investigations. In the other subset (*n* = 5), the two hippocampi of each brain were dissected out, homogenized, and further divided into two portions, then flash frozen in liquid nitrogen. After that, specimens were kept at − 80 °C. The first portion was used for enzyme-linked immunosorbent assay (ELISA) analysis of APC, Axin-1, TCF, BDNF, Dicer-1, β-catenin, c-Myc, and p-Erk while the second homogenate part was used for PCR analysis of miR-18 and miR-17-5p.

### Histopathology Processing

Tissue samples were flushed and fixed for 72 h in 10% neutral buffered formalin. All samples were trimmed then processed in serial grades of alcohols as well cleared in xylene. In addition, samples were infiltered, and a Paraplast tissue embedding media was used for embedding all samples. In addition, for demonstration of hippocampal subregions in different samples, rotatory microtome was used to obtain 5-μ thick sagittal brain sections. Finally, the sections were stained by hematoxylin and eosin as a general morphological examination staining method. All standard procedures for samples fixation and staining were conducted according to Culling [51].

### Immunohistochemistry

The hippocampal proliferative activity was measured using Ki-67 immunohistochemistry to elucidate β-catenin’s compensatory mechanism [52]. Tissue sections with a thickness of 5 μ were produced in paraffin, and the manufacturer’s protocol for immunohistochemistry was followed. Deparaffinized recovered tissue slices were treated with 0.3% H_2_O_2_ for 20 min. Then, at 4 °C overnight, brain samples were treated with Anti–ki67 (Cat. No. GTX16667, Genetex Co., USA, 1:100). Tissue sections were rinsed in phosphate buffered saline and treated with the secondary antibody HRP Envision kit (DAKO, USA) for 20 min before being washed and incubated with diaminobenzidine for 15 min. After that, tissue sections were washed by phosphate-buffered saline, and then hematoxylin counterstained, dehydrated, and cleared in xylene before being cover slid for microscopic analysis. Three random non-overlapping fields were selected and scanned from dentate gyrus (DG) regions of each sample for the determination of area-based percentage of immunoexpression levels of Ki-67 in immunohistochemically stained sections. All light microscopic examination and data were obtained using Leica Application module for histological analysis attached to full HD microscopic imaging system (Leica Microsystems GmbH, Germany) [53].

### Enzyme-Linked Immunosorbent Assays

Based on the instructions of the manufacturers, rat-specific ELISA kits were used. The following parameters were determined: Axin-1 (MyBiosource, San Diego, USA, Cat. No. MBS288517), APC (MyBiosource, San Diego, USA, Cat. No. MBS2702377), TCF (MyBiosource, San Diego, USA, Cat. No. MBS166467), Dicer-1 (MyBiosource, San Diego, USA, Cat. No. MBS2603565), β-catenin (MyBiosource, San Diego, USA, Cat. No. MBS843456), p-Erk (Thr183) (MyBiosource, San Diego, USA, Cat. No. MBS267200), c-Myc (MyBiosource, San Diego, USA, Cat. No. MBS2511134), and BDNF (MyBiosource, San Diego, USA, Cat. No. MBS824814). The results were expressed as nanogram per milligram of Axin-1, APC, TCF, and Dicer-1 and picogram per milligram of BDNF.

### Quantitative Real-Time PCR

After homogenization of brain tissues, extraction of total RNA was performed by mirvana kit (Thermo Fisher Scientific, USA, Cat No: A27828) according to the manufacture’s instruction. Quantification of the generated miRNA was measured by Nanodrop® spectrophotometer at 260 nm. The TaqMan® MicroRNA Assays are designed to detect and accurately quantify mature micRNAs using Applied Biosystems real-time PCR instruments. In the reverse transcription (RT) step, cDNA is reverse transcribed from miRNA samples using specific micRNA primers from the TaqMan® MicroRNA Assays and reagents from the TaqMan® MicroRNA Reverse Transcription Kit (Thermo Fisher Scientific, USA, Cat No: 4366596). Each 15-µL RT reaction consists of 7-µL master mix, 3-µL primer, and 5-µL miRNA sample. The master mix composed of 0.15 µL 100 mM dNTPs (with dTTP), 1 µL MultiScribe™ Reverse Transcriptase (Thermo Fisher Scientific, USA, Cat No: 4311235), 50 U/µL, 1.50 µL 10✕ Reverse Transcription Buffer, 0.19 µL RNase Inhibitor, and 20 U/µL and 4.16 µL nuclease-free water. Requirements for the amplification step comprised 10 min at 95 °C for promotion of AmpliTaq Gold DNA polymerase. Afterwards, denature stage with 40 cycles at 95 °C for 15 s after that annealing/extension phase at 60 °C for 1 min. Eventually, the expression of the chosen gene was normalized in reference to the mean critical threshold (CT) of the values of miR (U6) housekeeping gene expression using the ΔΔCt method [54]. Primer sequence for miR-17-5P and miR-18 gene was presented in Table 2.

**Table 2:** The primer sequence of the miR-17-5P and miR-18 genes

Gene symbol	Primer Sequence
miR-17-5p	Forward (F): 5′-AGCCCC GTCATAACAAAGGT-3′ Reverse (R): 5′-TCCTTTAGAAAA ACATTCAGCTAGG-3′
miR-18	Forward (F): 5′-CTGCGTGCTTTTTGTTCTAAGGT-3′ Reverse (R): 5′-CTTCTTATGCCAGAAGGAGCAC-3′
miR (U 6) House keeping	Forward (F): 5′-CTCGCTTCGGCAGCACA-3′ Reverse (R): 5′-AACGCTTCACGAATTTGCGT-3′

### Statistical Analysis

All data obtained were presented as mean ± S.D. Results were analyzed using one-way ANOVA, followed by Tukey’s multiple comparison tests. Data that did not meet normality were analyzed by Kruskal–Wallis one-way ANOVA on ranks, followed by Dunn’s post hoc test while those that did not meet homogeneity of variance were analyzed by Welch’s ANOVA, followed by Games-Howell test. Linear regression was performed for further investigating the relationship between anxious behavior and measured biochemical parameters (Pearson’s correlation and linear regression). Statistical analysis was performed using IBM SPSS Statistics Version 26 (IBM Corp., Armonk, New York). For all statistical tests, the level of significance was fixed at *P* < 0.05, and graphical design was presented by graph pad prism software Version 8 (San Diego, CA, USA).

## Results

### Vilazodone Mitigates Neurobehavioral Manifestations in Stressed Rats

**Fig. 2:**
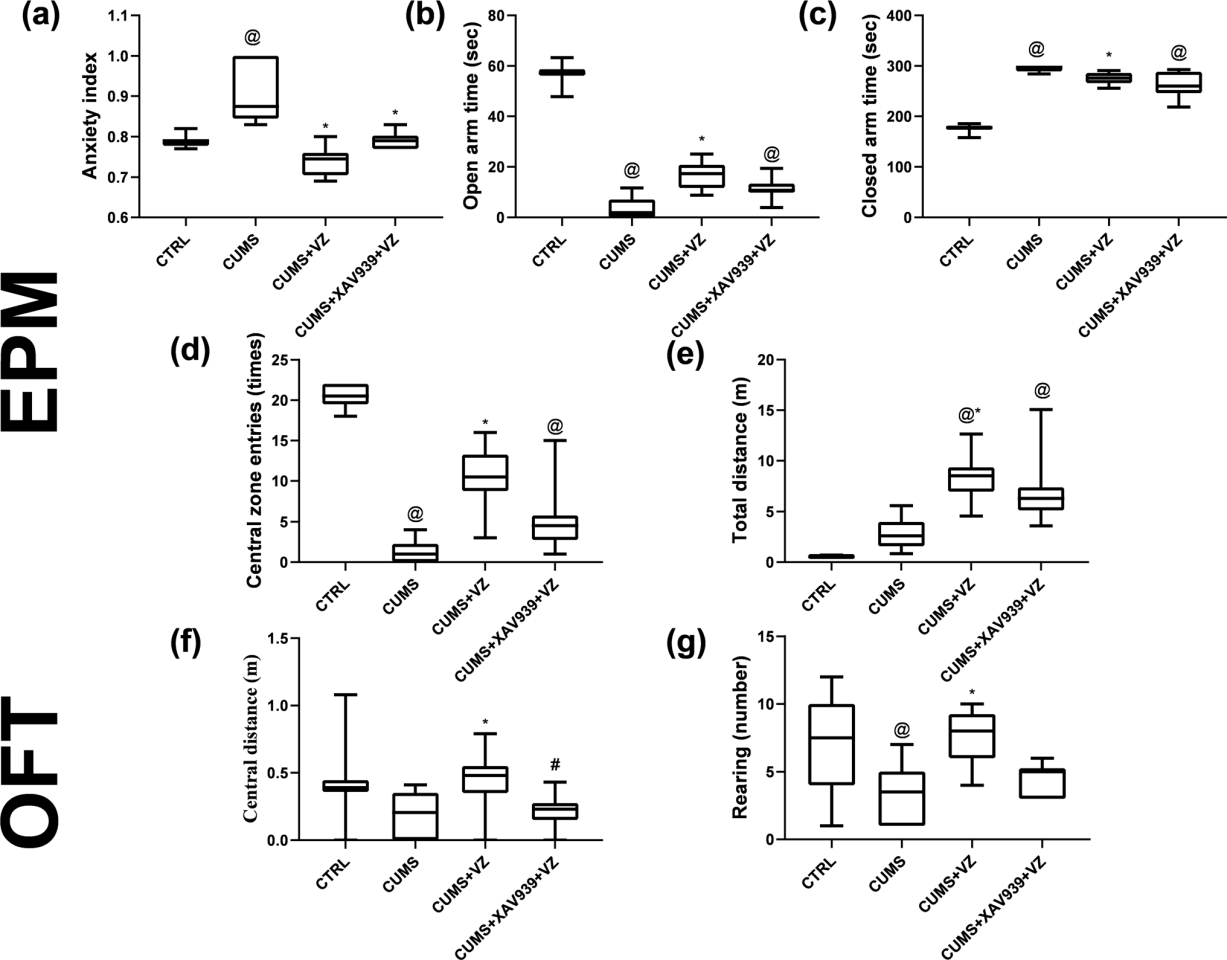
Effect of VZ on anxiety. Panels represent **a** anxiety index, **b** open-arm time, **c** closed-arm time, and **d** central zone entries and **e** total distance in EPM as well as OFT from **f** central distance and **g** rearing. Each bar with vertical line represents mean ± S.D. of 10 rats per group. Statistical analysis was performed using Kruskal–Wallis one-way ANOVA followed by Dunn’s post hoc test, *P* < 0.05, @vs CTRL, *vs CUMS, #vs CUMS + VZ

In EPM (Fig. 2), after exposure to the 21-day CUMS protocol, CUMS rats showed an increased anxiety index compared to CTRL group. In addition, CUMS rats showed reduced open-arm time and central zone entries along with increased closed-arm time. Treatment with VZ significantly decreased anxiety index compared to CUMS rats and consequently increased exploration toward open-arm time and central zone entries compared to CUMS group. Besides, VZ group showed decreased closed-arm time in comparison with CUMS one and total distance travelled. In OFT, CUMS rats significantly decreased rearing in comparison with CTRL group. Upon VZ administration, rearing and central distance increased compared to CUMS group. However, XAV939 rendered EPM open-arm time, closed-arm time, central zone entries, and total distance as well as OFT rearing and central distance like CUMS group on contrary to VZ group. On the other side, there was no sign of depression in SPT or FST in CUMS group.

### Vilazodone Alleviates Histopathological Alterations of Hippocampal CA2 and CA3 Regions Besides Restoring Normal Hippocampal Weight in CUMS rats

**Fig. 3:**
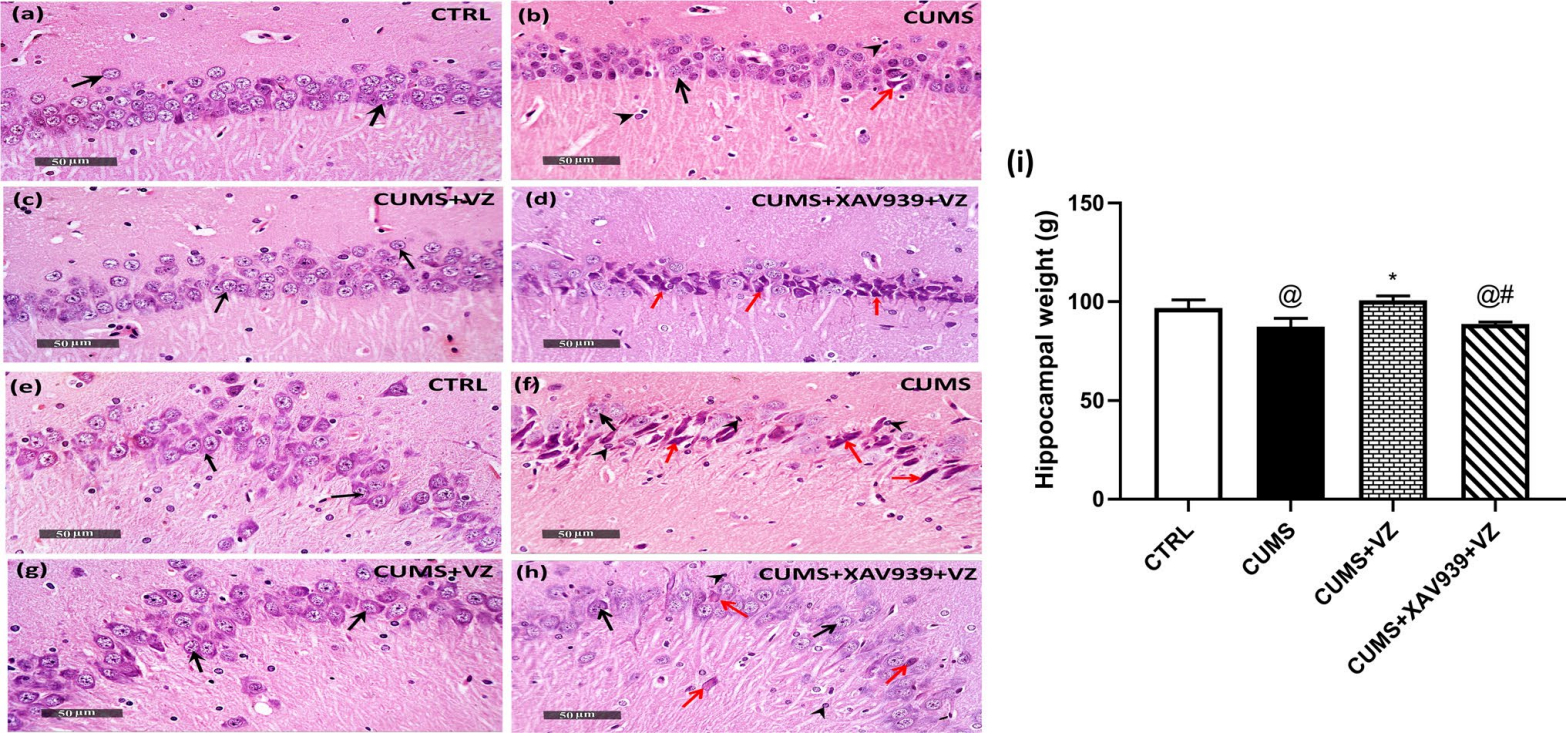
Effect of VZ and XAV939 on CA2 and CA3 histopathological regions and hippocampal weight in CUMS rat. Representative photomicrographs illustrating H&E staining of the hippocampus. Panels represent **a**, **e** CTRL group, **b**, **f** CUMS group, **c**, **g** CUMS + VZ group, and **d**, **h** CUMS + XAV939 + VZ group as well as **i** hippocampal weight. Black arrows indicate intact well-organized neurons, arrow heads indicate glial cell infiltration, and red arrows indicate degenerated neurons. Magnifications: × 400. Each bar with vertical line represents mean ± S.D (*n* = 3 in histological analysis and *n* = 6 in hippocampal weight). Statistical analysis was performed using one-way analysis of variance (ANOVA) followed by Tukey’s multiple comparison test, *P* < 0.05, @vs CTRL, *vs CUMS, #vs CUMS + VZ

Stress affects brain tissue histologically (Fig. 3). Herein, sections of both hippocampal CA2 and CA3 CTRL rats showed normal histological morphology with apparent intact polymorphic as well pyramidal neurons beside intact nuclear and subcellular details (black arrow). In CUMS group, CA2 and CA3 sections were accompanied with mild higher glial cell infiltrates (arrowhead). In addition, there were minimal records of degenerative neuronal changes (red arrow) with almost intact pyramidal neurons and intact subcellular details (black arrow). Furthermore, CUMS group exhibited reduction in hippocampal weight by 9.9% compared to CTRL one. Following VZ administration, hippocampi appeared with almost normal histological semblance in both CA2 and CA3 regions. In addition, VZ increased hippocampal weight by 15.4% in comparison with CUMS group. On the other side, XAV939 resembled CUMS and showed almost more hippocampal damage; there were moderate to severe records of hyperesenophilic, shrunken, and necrotic pyramidal neurons as well as few scattered degenerated neurons (red arrow) with less figures of apparent intact cells (black arrow). Furthermore, there are mild edema and vacuolization of brain matrix with mild glial cell infiltrates (arrowhead) as well as reduction in hippocampal weight (*F*_(3.2)_ = 24.12; *P* < 0.001) by 11.98% in comparison with VZ group.

### Vilazodone Restores Normal Dentate Gyrus Appearance With Balanced Immunohistochemical Examination

**Fig. 4:**
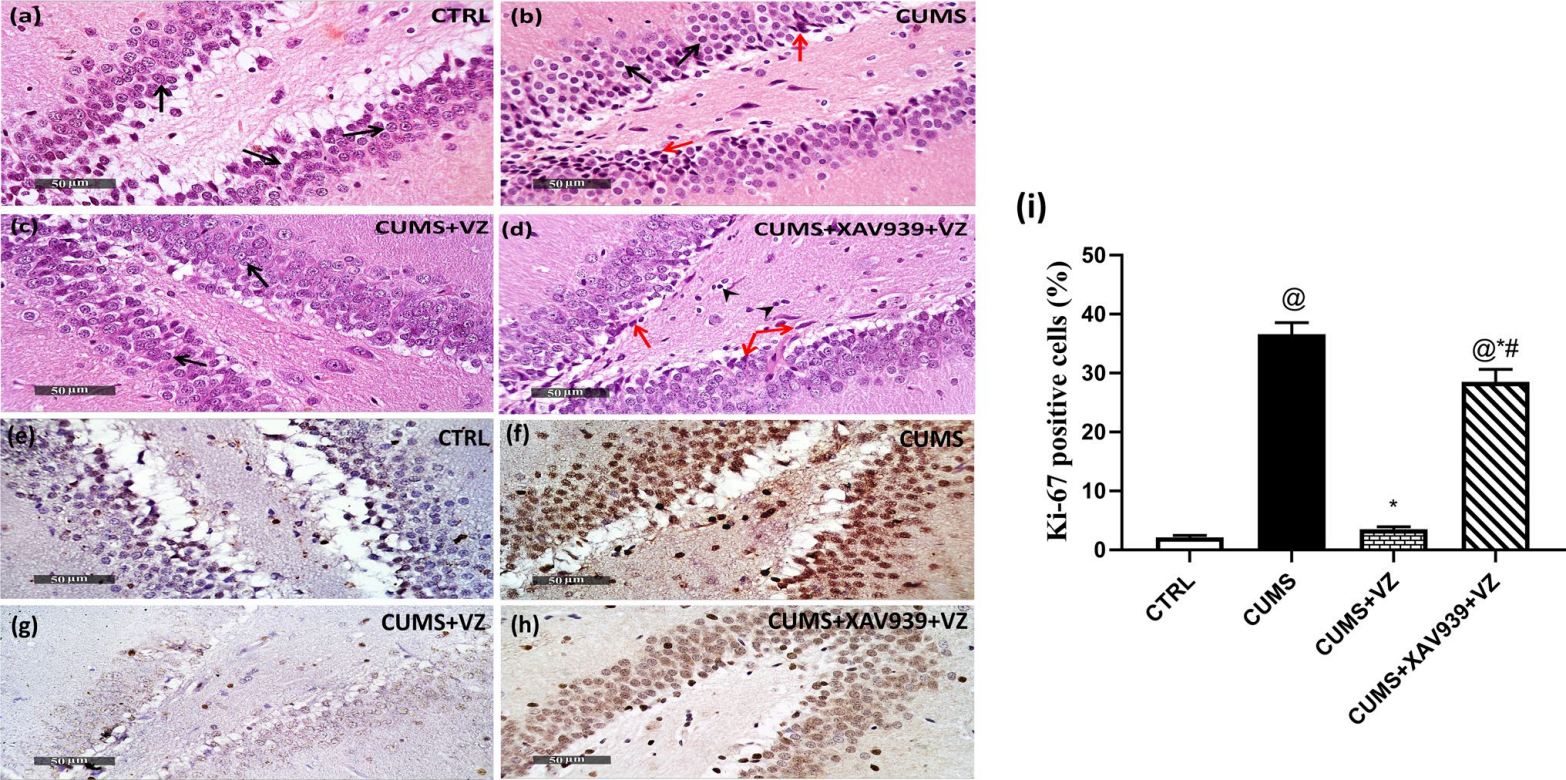
Effect of VZ and XAV939 on histopathological alteration in dentate gyrus region and Ki-67 immunohistochemical alterations in CUMS rat. Representative photomicrographs illustrating H&E staining of the hippocampal dentate gyrus from **a** CTRL group, **b** CUMS group, **c** CUMS + VZ, and **d** CUMS + XAV939 + VZ group as well as immunohistochemistry of Ki-67 from **e** CTRL, **f** CUMS, **g** CUMS + VZ and **h** CUMS + XAV939 + VZ groups besides **i** Ki-67 percentage. Black arrows indicate intact well-organized neurons, arrow heads indicate glial cell infiltration, and red arrows indicate degenerated neurons. Magnifications: × 400. Each bar with vertical line represents mean ± S.D (*n* = 3 in histological and immunohistochemical analysis). Statistical analysis was performed using one-way analysis of variance (ANOVA) followed by Tukey’s multiple comparison test, *P* < 0.05, @vs CTRL, *vs CUMS, #vs CUMS + VZ

In Fig. 4, DG of CTRL showed normal morphological features of hippocampal layers including granule cells at different zones with intact nuclear details (black arrow), intact hilar region with mild edema of brain matrix. Stressed hippocampi revealed many apparent intact granule cells with nuclear chromatin condensation (black arrow) and moderate records of intercellular edema and vacuolation of inner aspect of DG blades (star). Dentate gyrus upon vilazodone administration showed almost the same records as normal control samples. However, XAV939 section showed mild records of degenerated and necrotic inner small granule cells as well as hilar cells (red arrow) with moderate higher glial cells infiltrates (arrowhead). On the other side, stress compensatory mechanism elevated Ki-67 (*F*_(3.8)_ = 419.89; *P* < 0.001) percentage by 16.2-fold compared to CTRL while VZ declined this exaggerated response by 0.9-fold in comparison with CUMS group. However, XAV939 only decreased this exaggerated response by 0.2-fold compared to CUMS and 7.1-fold increase compared to VZ.

### Vilazodone Attenuates the Destructive Complex Activity Toward β-Catenin in Stressed Rats

**Fig. 5:**
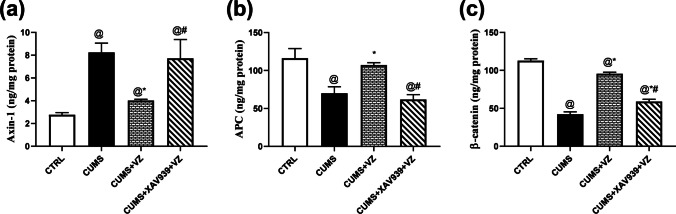
Effect of VZ on **a** Axin-1, **b** APC, and **c** β-catenin in CUMS rats. Each bar with vertical line represents mean ± S.D. of 5 rats per group. Statistics: **a** one-way ANOVA followed by Games-Howell’s post hoc test; **b**, **c** one-way analysis of variance (ANOVA) followed by Tukey’s multiple comparison test, *P* < 0.05, @vs CTRL, *vs CUMS, #vs CUMS + VZ

The destructive complex causes inhibition of β-catenin’s activity. Axin-1 and APC are components of this destructive complex. In Fig. 5, Axin-1 (*F*_(3.16)_ = 42.51; *P* < 0.001) increased approximately twofold in CUMS group compared to the CTRL one. On the other side, VZ suppressed the increased Axin-1 by 51.3% while XAV939 resembled CUMS and negated 92.1% of VZ action. On the contrary, stress decreased APC signal (*F*_(3.16)_ = 51.31; *P* < 0.001) by 39.7%, which was restored by VZ by 53% increase in APC in comparison to CUMS rats. However, XAV939 resembled CUMS and annulled 42.3% of VZ action and rendered this signal as observed in the model group. In accordance, β-catenin (*F*_(3.16)_ = 722.08; *P* < 0.001) protein expression decreased by 62.5% in CUMS group while VZ improved it by 112.6%. However, XAV939 just increased β-catenin by 39.5% and negated 38.2% of VZ action on β-catenin which worsened the case and made this group like the model one.

### Vilazodone Modulates the Downstream Targets of β-Catenin in Stressed Rats

**Fig. 6:**
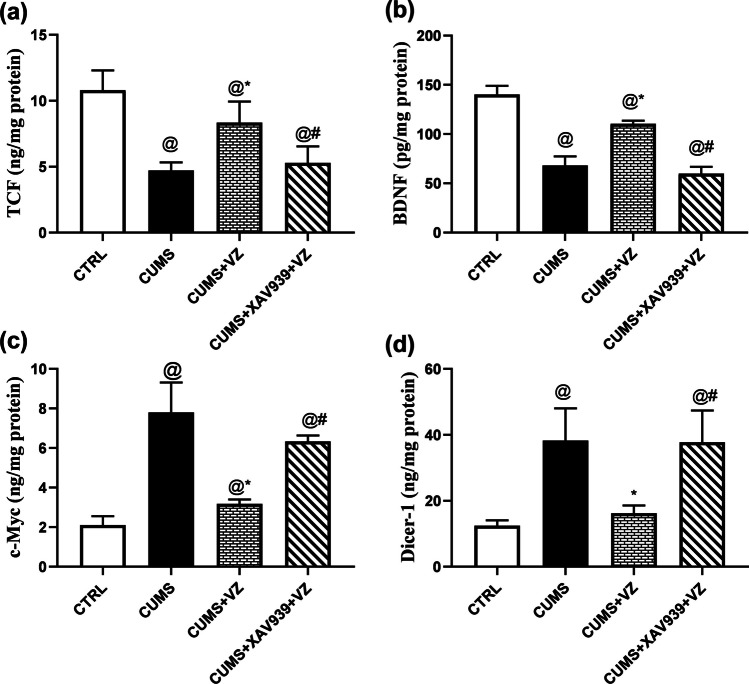
Effect of VZ on **a** TCF, **b** BDNF, **c** c-myc, and **d** Dicer-1 in CUMS rats. Each bar with vertical line represents mean ± S.D. of five rats per group. Statistics: **a**, **b** and **d** one-way ANOVA followed by Tukey’s multiple comparison test; **c** one-way ANOVA followed by Games-Howell’s post hoc test, *P* < 0.05, @vs CTRL, *vs CUMS, #vs CUMS + VZ

In Fig. 6, CUMS caused 56.4% reduction of TCF signal (*F*_(3.16)_ = 23.74; *P* < 0.001), while VZ increased this signal by 76.8%. However, XAV939 annulled 36.5% of VZ action, which rendered this signal as observed in the model group. In addition, stress reduced BDNF signal (*F*_(3.16)_ = 129.76; *P* < 0.001) by 51.3%, while VZ boosted BDNF signal by 62%. However, XAV939 reduced VZ signal by 45.9% and returned this signal to CUMS. On the other side, c-Myc (*F*_(3.16)_ = 53.93; *P* < 0.001) increased significantly in CUMS group by 2.7-fold, and when VZ was given, the increased expression of c-Myc decreased significantly by 0.6-fold compared to CUMS group. However, XAV939 significantly increased c-Myc expression by 0.99-fold compared to VZ which returned c-Myc expression to stressed state. Concomitantly, there was a 2.1-fold increase in Dicer-1 signal (*F*_(3.16)_ = 19.44; *P* < 0.001) within stress condition and a 57.6% reduction upon VZ administration that returned this signal to normal condition. When XAV939 was utilized, Dicer-1 returned to reach that of CUMS group.

### Vilazodone Relieves the Flaring of the Compensatory Mechanism: miR-17-5p, miR-18, and p-Erk in Stressed Rats

**Fig. 7:**
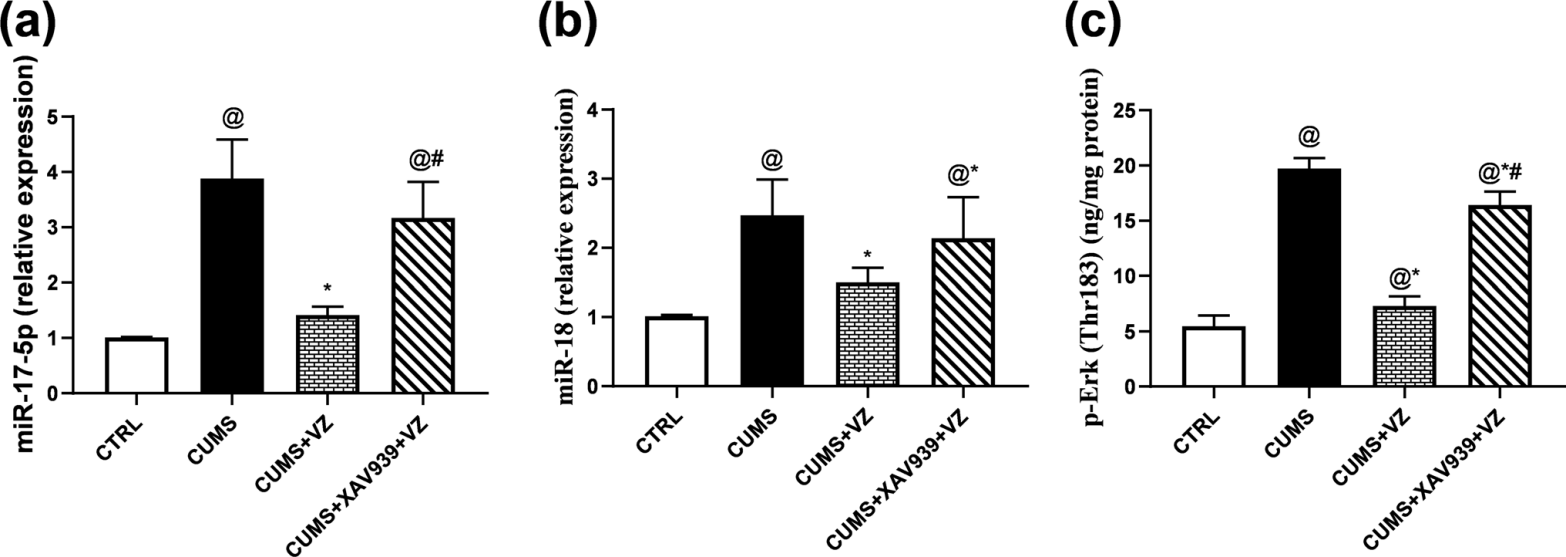
Effect of VZ on **a** miR-17-5p, **b** miR-18, **c** p-Erk in CUMS rats. Each bar with vertical line represents mean ± S.D. of five rats per group, using one-way ANOVA followed by Tukey’s multiple comparison test, *P* < 0.05, @vs CTRL, *vs CUMS, #vs CUMS + VZ

In Fig. 7, miR-17-5p (*F*_(3.16)_ = 40.03; *P* < 0.001) was significantly raised by 2.8-fold in CUMS group compared to CTRL one. Treatment with VZ significantly suppressed the overdrawn miR-17-5p expression by 63.4%. Such suppression was reversed by the 1.2-fold increase after XAV939 administration which resembled CUMS. Furthermore, miR-18 (*F*_(3.16)_ = 12.72; *P* < 0.001) increased by 1.43-fold in CUMS group in comparison with control group while VZ caused 39.3% reduction of its flare to resemble CTRL group. However, XAV939 just causes 13.4% increase in this signal compared to CUMS group. In addition, p-Erk (*F*_(3.16)_ = 234.70; *P* < 0.001) was significantly increased in the model by 2.6-fold while VZ efficiently decreased it by 63% to normalize. On contrary, XAV939 just decreased this signal by 0.17-fold compared to CUMS and caused 1.2-fold rise compared to VZ which avoided reaching the normal level.

### Linear Regression Model Showing the Correlation Between Anxiety-Like Behavior and Neurogenesis-Related Markers

**Fig. 8:**
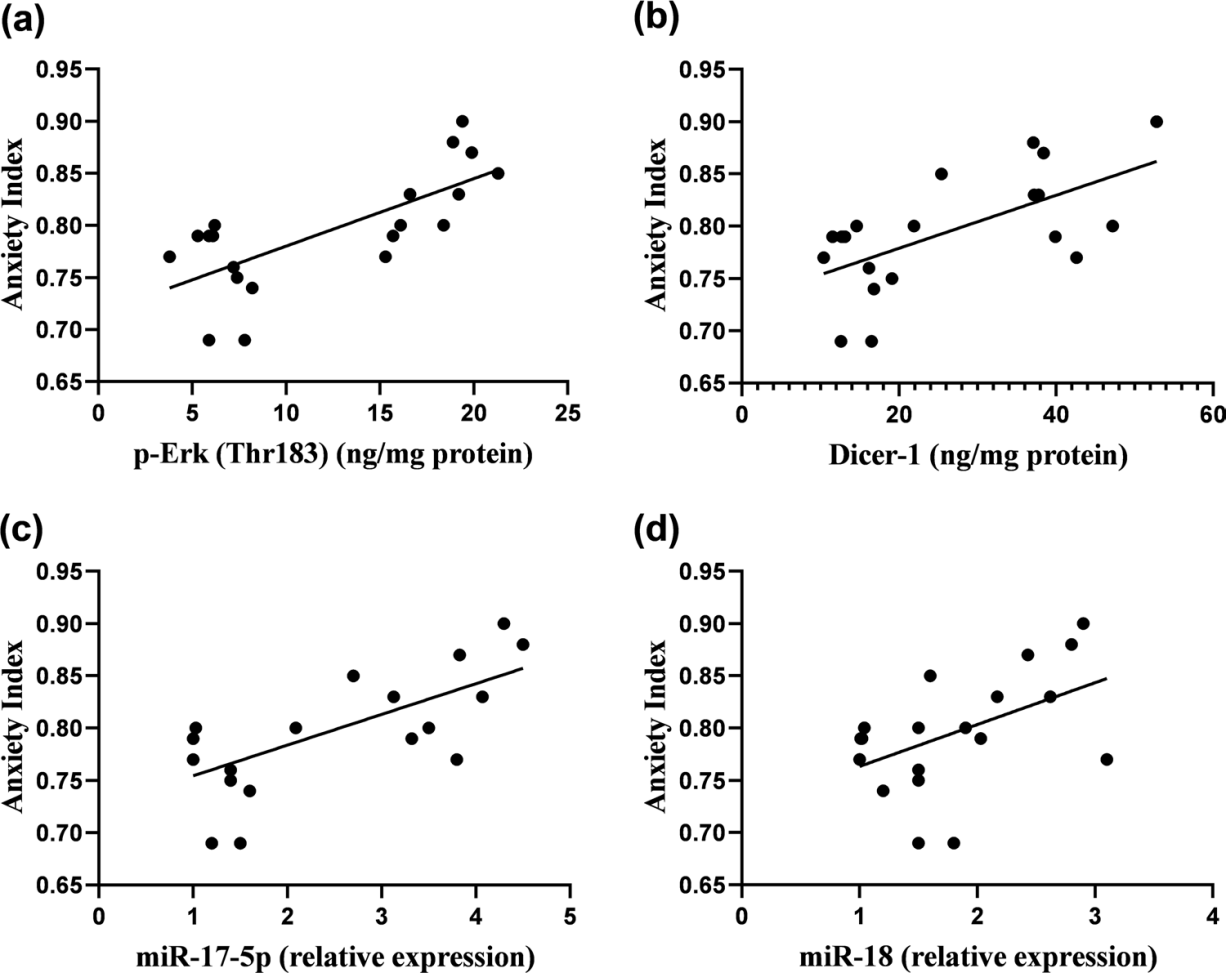
Linear regression model showing the correlation between anxiety index and **a** p-Erk, **b** Dicer-1, **c** miR-17-5p, **d** miR-18, using Pearson’s correlation and linear regression, *P* < 0.05

**Fig. 9:**
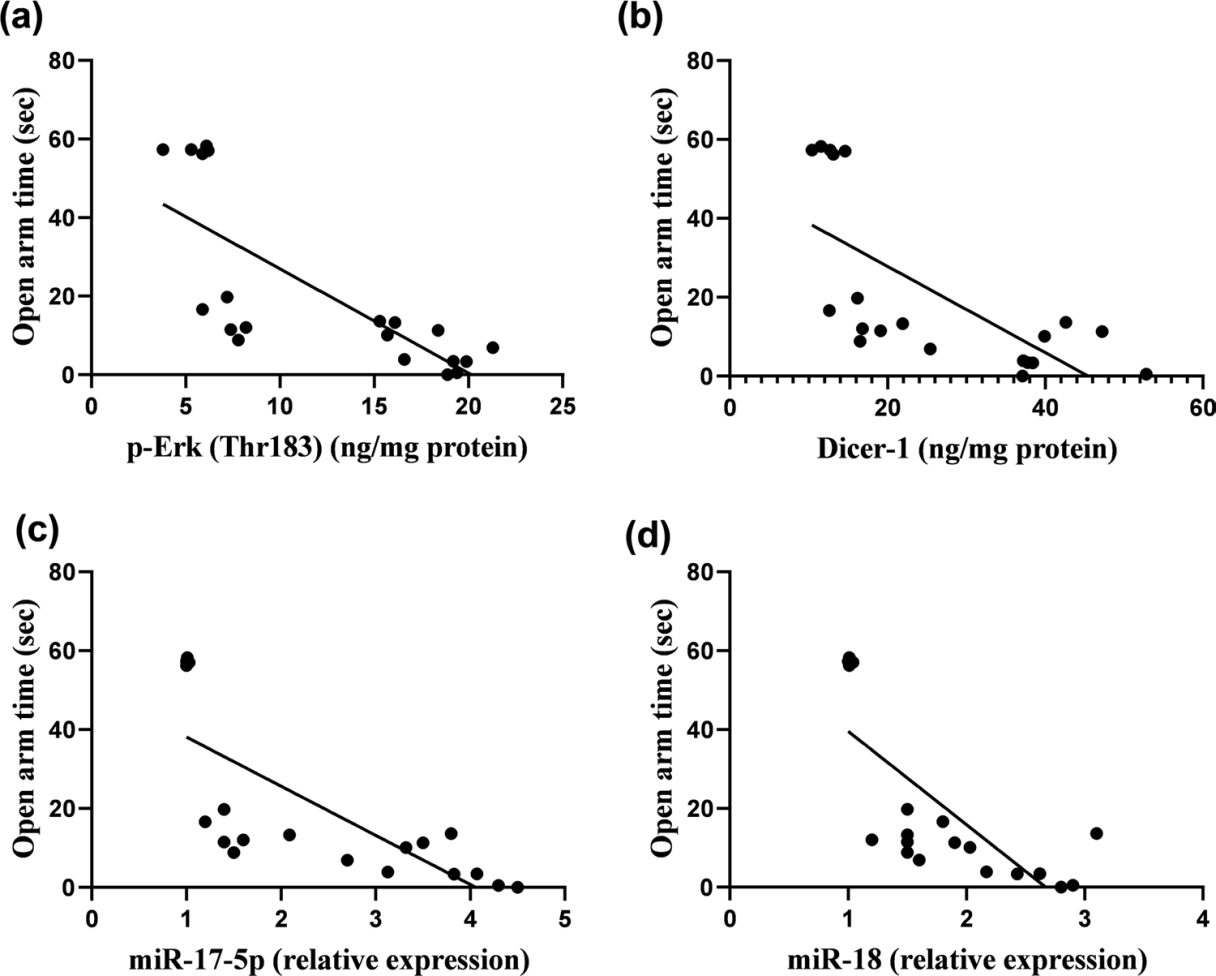
Linear regression model showing the correlation between open arm time and **a** p-Erk, **b** Dicer-1, **c** miR-17-5p, **d** miR-18, using Pearson’s correlation and linear regression, *P* < 0.05

**Fig. 10:**
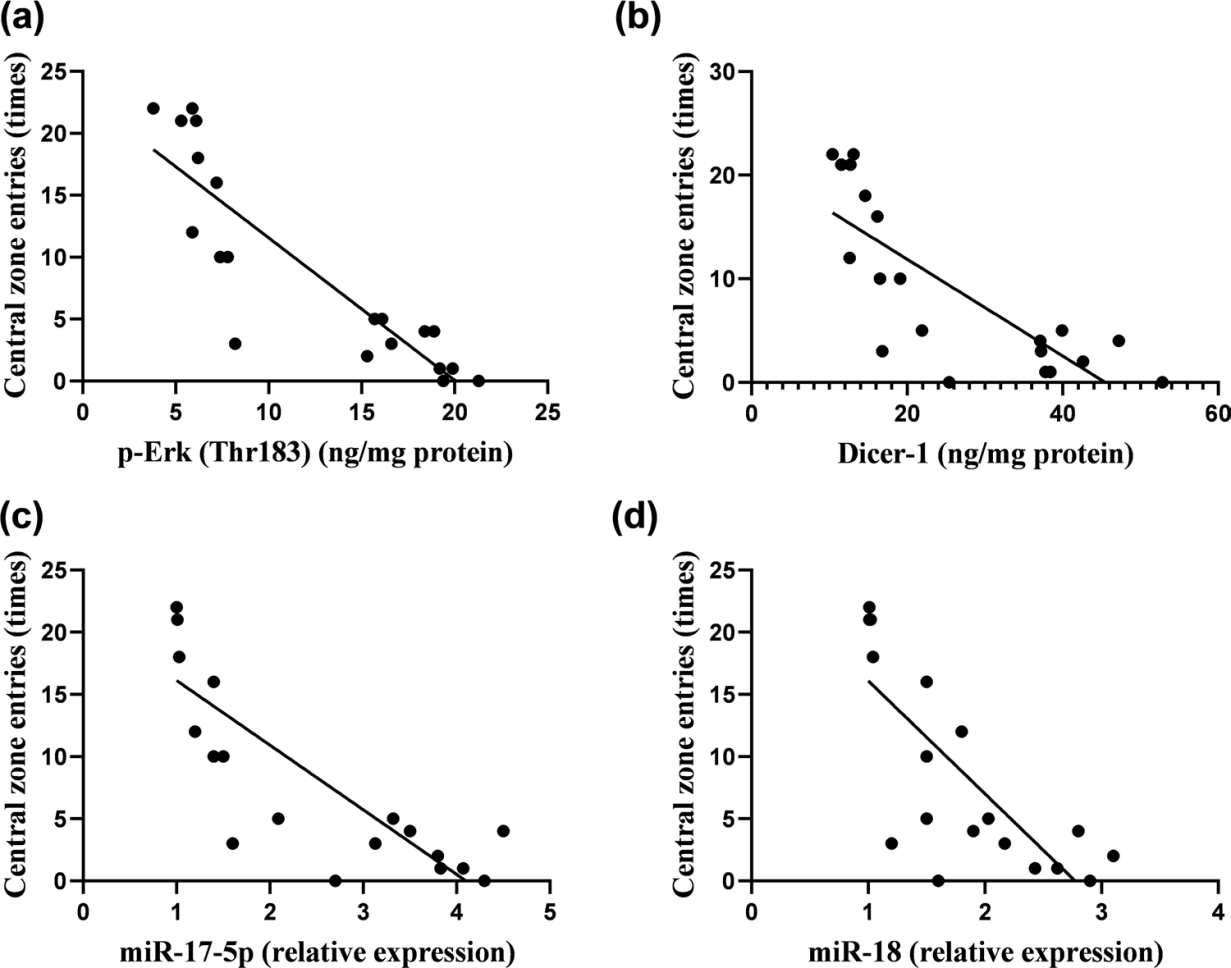
Linear regression model showing the correlation between central zone entries and **a** p-Erk, **b** Dicer-1, **c** miR-17-5p, **d** miR-18, using Pearson’s correlation and linear regression, *P* < 0.05

In Fig. 8, anxiety index is positively correlated with p-Erk (*r* = 0.723, *P* < 0.001), Dicer-1 (*r* = 0.627, *P* < 0.01), miR-17-5p (*r* = 0.685, *P* < 0.001), and miR-18 (*r* = 0.494, *P* < 0.05). In Fig. 9, open-arm time is negatively correlated with p-Erk (*r* =  − 0.750, *P* < 0.001), Dicer-1 (*r* =  − 0.686, *P* < 0.001), miR-17-5p (*r* =  − 0.737, *P* < 0.001), and miR-18 (*r* =  − 0.740, *P* < 0.001). In Fig. 10, central zone entries are negatively correlated with p-Erk (*r* =  − 0.879, *P* < 0.001), Dicer-1 (*r* =  − 0.791, *P* < 0.001), miR-17-5p (*r* =  − 0.830, *P* < 0.001), and miR-18 (*r* =  − 0.767, *P* < 0.001). Multiple regression of anxiety index (*R*^2^ = 0.608), open-arm time (*R*^2^ = 0.665), and central zone entries (*R*^2^ = 0.810) are displayed in Tables 3, 4, and 5, respectively.

**Table 3:** Multiple linear models between anxiety index and different neurogenesis related markers

	Unstandardized Coefficients		Standardized coefficients	*t*	Sig
	B	Std. Error	Beta		
(Constant)	.768	.036		21.584	.000
p-Erk	.001	.004	.165	.351	.731
Dicer-1	− .001	.002	− .253	− .497	.627
miR-17-5p	.067	.041	1.563	1.637	.122
miR-18	− .069	.040	− .855	− 1.742	.102

**Table 4:** Multiple linear models between open-arm time and different neurogenesis-related markers

	Unstandardized coefficients		Standardized coefficients	*t*	Sig
	B	Std. Error	Beta		
(Constant)	74.128	12.972		5.715	.000
p-Erk	− 3.210	1.539	− .909	− 2.086	.054
Dicer-1	.060	.750	.038	.080	.937
miR-17-5p	14.963	14.925	.884	1.003	.332
miR-18	− 28.541	14.511	− .893	− 1.967	.068

**Table 5:** Multiple linear models between central zone entries and different neurogenesis related markers

	Unstandardized Coefficients		Standardized coefficients	*t*	Sig
	B	Std. Error	Beta		
(Constant)	27.975	3.623		7.722	.000
p-Erk	− 1.323	.430	− 1.012	− 3.078	.008
Dicer-1	− .069	.209	− .117	− .331	.746
miR-17-5p	4.491	4.168	.717	1.077	.298
miR-18	− 6.525	4.052	− .551	− 1.610	.128

## Discussion

The present study revealed a novel role of VZ in dealing with the anxious rats with the power to untangle some of the ambiguity regarding β-catenin pathway by (i) reduction of anxious behavior manifestations; (ii) improvement of the histological appearance; (iii) reduced activity of the destructive complex by decreasing Axin-1 and increasing APC signals; (iv) elevation of the reduced β-catenin; with improvement of TCF and BDNF signals as downstream targets of β-catenin; (v) modulation of the exaggerated compensatory mechanism of p-Erk, c-Myc, Dicer-1, miR-17-5p, and miR-18; (vi) clarifying APC’s multifaceted role in the destructive complex as a supporter of β-catenin within β-catenin compensated action; (vii) TCF and BDNF may be stronger β-catenin followers than Dicer-1 or c-Myc, in contrast to p-Erk.

Hippocampal neurogenesis is one of the fundamental mechanisms elucidating the etiology of this burdensome disorder. Though increased neurogenesis may implicate in deposition of anxiety, impaired neurogenesis process plays a key role in precipitating psychiatric diseases like depression [40, 55]. It is observed that β-catenin dysfunction is linked to neurogenesis and neurotransmitter release [56, 57]. Herein, reduced β-catenin may speculate raised neuron sprouting, as correlated by the highest Ki-67 expression in CUMS group. This value supports the role of diminished β-catenin in eliciting compensatory mechanism which modifies the activity of positive and negative pathway regulators [39]. Herein, boosting neurogenesis encountered pros and cons. After 3 weeks of CUMS, depression did not appear in SPT or FST where neurogenesis gained fruitful protection against depression, and this agrees with Carrard et al. [58]. Moreover, this result was in agreement with the neuroplasticity hypothesis as it supported the positive correlation between antidepressant action and neurogenesis [59]. On the contrary, it encountered negative consequences of precipitating anxiety. Researchers supported the influence of increased neurogenesis in precipitating anxiety and protecting against depression [60]. Indeed, CUMS is a robust animal model for depression [61] and was supposed to cause depression in this study. However, Xiang et al. showed that some animals may respond while others may not respond at all [62]. Even same species exposed to the same CUMS may exhibit different depressive susceptibility as in adult male Sprague Dawley rats [63]. In accordance, sucrose preference in male Wistar rats decreased after the first week of 32 days of chronic stress and may disappear by the end of the third week, indicating animal adaptation to stressful factors [64]. In addition, chronic mild stress for 5 weeks in male Wistar rats did not exhibit any difference compared to control group in sucrose preference test [65].

Together, chronic unpredictable mild stress for 3 weeks in mice can result in anxiety rather than depression [66]. Herein, CUMS induced anxiety in EPM and OFT rather than depression in SPT or FST which correlates favorably with Yu et al. [67] and El-Kadi et al. [68].

Herein, CUMS group showed increased anxiety index and decreased exploration time in open arms supported by decreased central zone entries and exhibited the maximum closed-arm time in addition to the least rearing. According to Cunniff et al. [69], anxious rats preferred to stay in familiar or safe areas and avoided open regions. Herein, VZ increased open-arms time, total distance travelled, and central zone entries as well as decreased anxiety index, central distance, and rearing. Indeed, decreased anxiety index is pointed as an indicative of increased exploration activity in open arms and supported the anxiolytic activity of VZ. The effects of VZ are counteracted by XAV939 administration where open-arm time, closed-arm time, central zone entries, and total distance were returned to values of the stressed state. In contrast, Adamec et al. [70] displayed that VZ, given at a single therapeutic or prophylactic dose of 10 mg/kg to stressed male Long–Evans hooded rats, was anxiogenic in the startle stress test while it did not affect anxiety assessments in the plus-maze test. These effects may differ from current results where Adamec et al. used acute administration of VZ to male rats using cats as a predator stress model [70]. The difference in animals, sex, stress paradigm, and exposure time may influence the therapeutic outcomes. In addition, this discrepancy from current results may be owing to the fact that acute administration of antidepressants is anxiogenic, while continuous maintenance doses are anxiolytic [71].

Hippocampal neurogenesis may be a critical factor in the link between stress, anxiety and depression-like behaviors [72]. Hippocampal DG and subventricular zone are two restricted regions for continuous neurogenesis. However, the hippocampus rather than the subventricular zone is pivotal for mood regulation, which implicates anxiety and depression disorders [20, 73]. Consequently, hippocampus is considered a distinct specialized microenvironment as it is highly concerned with neurogenesis rather than other brain parts [74]. Herein, VZ normalized Ki-67 expression in DG and possibly subsided the exaggerated load of the neurogenesis stimulating factors. Within only 2 weeks, VZ increased β-catenin, and its downstream targets in turn regained normal hippocampal weight. This may be supported by the work of Tunc-Ozcan et al. [75] in which VZ increased neurogenesis in a depression model. On the other side, blocking β-catenin by XAV939 put further stress and returned hippocampal weight and other exaggerated load of c-Myc, Dicer-1, and miR-17-5p resembling CUMS as noted by high Ki-67 percentage. In the current study, CA3 exhibited more histological deterioration in CUMS. This may be explained by Pang et al. [76] as CA3 is highly influenced by β-catenin expression and by Rosiles et al. [77] where CA2 exhibits higher resilience toward death. Moreover, VZ almost improved CA2 and CA3 histological representation; however, XAV939 increased stress burden. Despite the prominence of neurogenesis in CUMS and XAV939-treated groups, such effect was not enough to hinder the histological alterations.

Axin-1 and APC are together responsible for degradation of β-catenin. This study is in partial agreement with Silva et al. [78] as Axin-1 dropped β-catenin with subsequent reduction in TCF in both CUMS and XAV939 groups. However, the current study demonstrated elevated APC rather than previously documented. This finding has been pointed by Parker and Neufeld [79] and Takacs et al. [80]. The current study supported Parker and Neufeld [79] rather than Silva et al. [78] regarding APC role in the destructive complex as this affirmed the role of APC in the destructive complex in favor of β-catenin activation rather than its destruction. In addition, the decline in β-catenin expression following enhanced Axin-1 signal in XAV939 group was confirmed by Alula et al. [81] as XAV939 has dual capacity of stabilizing Axin-1 protein and increasing β-catenin degradation. Interestingly, VZ has salutary action in boosting β-catenin signal in part through reducing the activity of the destructive complex by its action on decreasing Axin-1 and flourishing APC signals in addition to enrichment of TCF and BDNF signals. These effects returned to stressed state after XAV939. This value correlates favorably with this study and further supports the strong dependency of TCF and BDNF toward β-catenin signal. Many researchers discussed the importance of BDNF in induction of neurogenesis; however, in the present study, BDNF was lower in the CUMS and XAV939-treated groups. This may be owed to consumption of BDNF to display plasticity and to negate to the least some effects of the stress. BDNF is a plasticity facilitator, and the result of its deficiency can be detrimental or beneficial in some cases like depression recovery which appeared in absence of depression in both FST and SPT in CUMS group, is in agreement with Karatsoreos and McEwen [82]. Noteworthy, the levels of BDNF and its receptor are crucial in depression pathogenesis. The neurotrophin theory is important in the interpretation of antidepressant effects. However, BDNF activation and its TrkB receptor can independently govern the therapeutic benefits of traditional antidepressants. It is found that BDNF may not be incorporated in antidepressant-induced neurogenesis [83]. Antidepressant properties can be attained by transactivating TrkB, in absence of endogenous BDNF, via glucocorticoids, kainic acid, and anandamide, an endocannabinoid [84]. In accordance, Gheorghe et al. [85] revealed that antidepressant efficacy is dependent on BDNF downstream signaling rather than BDNF itself. In Kv1.1 knock-out mice, TrkB receptors were activated by mechanisms other than the neurotrophins, where TrkB signaling was elevated in BDNF-independent manner [86]. Together, neurogenesis is related strongly to BDNF; however, studies illustrated that neurogenesis may be independent of BDNF. Ketamine as an antidepressant enhanced neurogenesis in BDNF/TrkB-dependent and independent behavior [87]. Besides, intraventricular administration of BDNF to male Sprague Dawley rats diminished neurogenesis [88]. Ferreira et al. attributed the hippocampal plasticity and neurogenesis to high corticosterone levels independent of BDNF overexpression [89]. As well, another type of TrkB receptor, truncated TrkB receptor, showed negative impact on full-length trkB receptor which repressed its neurogenic character [90]. TrkB, TrkC, and BDNF receptors undergo activation by epidermal growth factor receptor signaling rather than by BDNF, which may enhance neurogenesis via p-Erk [91]. Thus, these previous findings may interpret the VZ-associated neurogenesis in presence of BDNF recession.

The transcription factor c-Myc controls the expression of more than 15% of the entire genome and influences cell cycle genes, cell adhesion, viability, protein synthesis, and neurogenesis. Together, Dicer-1, a cellular micro-RNA generating protein, plays a significant role in neuronal differentiation and proliferation. Herein, c-Myc and Dicer-1 were upregulated in CUMS group in an attempt of the body to cope with stress; however, anxiety progressed. Vilazodone suppressed both flared signals while administration of XAV939 exaggerated both. Even though Dicer-1 and c-Myc are downstream targets of β-catenin, there was an increase of both in the presence of a reduced β-catenin signal in CUMS group. In addition, c-Myc and Dicer-1 were supposed to decline upon XAV939 administration as XAV939 blocks β-catenin downstream targets. This apparently displayed that c-Myc and Dicer-1 did not depend on β-catenin. This finding may be attributed to the reduced APC signal that may contribute to this unexpected activation of c-Myc as observed previously by Finch et al. and Lee et al. [92, 93]. Although c-Myc is a downstream target for β-catenin, reduction of β-catenin did not influence c-Myc that is still boosted. Therefore, there was a stronger signal that could trigger this activation rather than β-catenin. Regarding this unexpected result, the inverse relationship between c-Myc and β-catenin was observed in two different conditions: subclinical hypothyroidism and basal-like breast cancer [94, 95]. These were interpreted by Zuo et al. [96] and de Rooij et al. [97] who found activated Erk signal is the upstream mediator of c-Myc and Dicer-1 activation, respectively. Consequently, Erk may be the common cue where c-Myc and Dicer-1 can be activated either by β-catenin or p-Erk.

Of note, Erk regulates major cellular functions in multiple neuropsychiatric disorders including neurogenesis. Herein, p-Erk increased significantly in the presence of a reciprocal relationship between c-Myc and β-catenin in CUMS group, which may reconcile some of these unexpected results. Furthermore, according to Kurokawa et al. [98], Erk contributes to stress coping or adaptation. Thus, p-Erk may be part of the compensatory mechanism managed by β-catenin. In addition, Zhang and Hashimoto [99] support the antidepressant effect of Erk while Michalak et al. [100] support its anxiogenic activity. The absence of depression in SPT or FST in CUMS group at the end of the study supported the anti-depressive character. However, the anxiogenic activity was supported by EPM test. In accordance with c-Myc, the results of Dicer-1 may be explained by this way as both are under the power of p-Erk [101, 102]. Thus, p-Erk may be the chief regulator of the neurogenesis process by exaggerated c-Myc and Dicer-1. Consequently, c-Myc and Dicer-1 may follow a stronger signal, and this strong signal may be p-Erk; hence, they might be considered indirect followers of β-catenin. Ultimately, TCF is a stronger follower for β-catenin with weaker activity regarding Erk. In addition, c-Myc and Dicer-1 may be weaker followers of β-catenin and stronger ones regarding p-Erk. Based on the previous findings, it was most likely that decreased β-catenin signal triggered novel compensatory mechanism managed by p-Erk that precipitates anxiety. Thus, p-Erk is thought to be the primary cause of the anxious stress by the possibility of enrichment of neurogenesis-stimulating factors which implicated anxiety. In the present study, VZ decreased the exaggerated effect of the increased Erk’s downstream targets, which may be by removing the tension of the dropped β-catenin and giving chance to the over-expressed c-Myc, Dicer-1, and other neurogenesis-stimulating factor to decline.

The miR-17–92 family can rise or decline in anxiety and be directed through c-Myc and Dicer-1 [103, 104]. Herein, both miR-17-5p and miR-18 were boosted in CUMS group in an epigenomic regulation of the body to cope with the applied stress. This is in agreement with Merril et al. [105] as epigenomic regulation is considered as a part of the adaptive response from the genome toward external environment. Consequently, it is hypothesized that a high expression of both increased neurogenesis threshold, contributing to antidepressant and anxious effects. Herein, this study highlighted the gap between protection against depression and vulnerability toward anxiety. Vilazodone reduced the flare of both miRNAs. miR-17-5p may follow the pattern of both c-Myc and Dicer-1 in which they decreased with VZ and increased after administration of XAV939. However, both were considered as targets of β-catenin that does not apparently follow all the time. This may be considered a cue that mir-17-5p may go through this way.

β-catenin’s compensatory mechanism is involved in allostasis that promoted protection from depression, where allostasis is a biological response that promotes adaptation. This agrees with Karatsoreos and McEwen [82] and further supports the role of β-catenin’s compensatory mechanism in implication of allostatic load. Consequently, exaggerated neurogenesis threshold accounted for this allostatic load that paved to anxiety appearance. This is presented in the current study where positive correlation was observed between anxiety-like behavior with the compensated neurogenesis stimulating factors: p-Erk, Dicer-1, miR-17-5p, and miR-18. Collectively, these results indicated a unique pathophysiology of anxiety. Advantageously, VZ efficiently raised β-catenin signal which was the leading cause of eliciting this compensatory mechanism and modulated the increased allostatic load of p-Erk, c-Myc, and Dicer-1 besides miR-17-5p and miR-18. In addition, it potentially reduced activity of the destructive complex by decreasing axin-1 and enhancing APC signals. This current approach can be a pioneering theme to enhance our understanding of VZ’s role in the treatment of anxiety through β-catenin signaling. This basic research can be applied clinically by investigating the power of stress in group toward stress buffering which appeared in absence of depression. Moreover, the current study is considered as a starting point toward studying clinically the effect of VZ on neurogenesis-induced anxious patient as well as tailoring drugs that relies on β-catenin signaling, focusing on the primary cause of decreased β-catenin signaling itself rather than the secondary cause of its allostatic load of increasing Erk, c-Myc, and miR-17-5p and miR-18. Monitoring of estrous cycle and studying the effect of VZ on other activators like EGFR, Akt, and STAT3 in addition to NOTCH pathway is considered as a limitation in this study. Regarding future studies in the prospect of this area, it would be appealing to block p-Erk either by the Ang II type 1 receptor antagonist, fluorofenidone, or another signal inhibitor to clarify if p-Erk is the main responder for mir-18 activation. In addition, changing the model’s sphere and studying the manipulation of internal or external factors controlling stress response like gender, age, genetic, and stress duration as well as stress type is essential. Furthermore, implementing methods to monitor the estrous cycle of females like hormone measurements and vaginal cytology to control the influence of estrous cycle is recommended.

Finally, to the best of our knowledge, the current study presented the first report for the novel anxiolytic effect of VZ that was manifested by preventing anxiety manifestations via raising β-catenin signaling and modulation of the exaggerated maladaptive response. Thus, this is offering a new perspective for the potential role of VZ in the treatment of anxiety.

## Data Availability

The datasets generated during and/or analyzed during the current study are available from the corresponding author, upon reasonable request.

## Abbreviations

miRNAs: MicroRNAs
CTRL: Control group
CUMS: Chronic unpredictable mild stress
BDNF: Brain-derived neurotrophic factor
ELISA: Enzyme-linked immunosorbent assay
APC: Adenomatous polyposis coli
AU: Arbitrary units
CA: Cornu ammonis
DG: Dentate gyrus
EPM: Elevated plus maze
OFT: Open field test
p-Erk: Phosphorylated extracellular signal- regulated kinases
SPT: Sucrose preference test
FST: Forced swimming test
SSRI: Selective serotonin reuptake inhibitor
TCF: T cell factor
VZ: Vilazodone
